# An Isoxazole Chalcone Derivative Enhances Melanogenesis in B16 Melanoma Cells via the Akt/GSK3β/β-Catenin Signaling Pathways

**DOI:** 10.3390/molecules22122077

**Published:** 2017-11-28

**Authors:** Li Yin, Chao Niu, Li-xin Liao, Jun Dou, Maidina Habasi, Haji Akber Aisa

**Affiliations:** 1 Key Laboratory of Plant Resources and Chemistry of Arid Zone, Xinjiang Technical Institute of Physics and Chemistry, Chinese Academy of Sciences, Urumqi 830011, Xinjiang, China; jinshiyi2003@163.com (L.Y.); niuchao@ms.xjb.ac.cn (C.N.); doujun@ms.xjb.ac.cn (J.D.); maidn@ms.xjb.ac.cn (M.K.); 2 Department of Chemical Engineering, Xinjiang Institutes of Engineering, Urumqi 830091, Xinjiang, China; lixinliao@hotmail.com; 3 University of the Chinese Academy of Sciences, Beijing 100039, China

**Keywords:** chalcone derivative, PI3K/Akt, Wnt/β-catenin, melanogenesis, B16 melanoma

## Abstract

Plants or plant-derived products have been routinely used in several traditional medicine systems for vitiligo treatment. It is well-known that melanogenesis can be promoted by certain flavonoid compounds isolated from the traditional Uyghur medicinal plant, Kaliziri. Therefore, Chalcones, one class of flavonoid compounds, has become an interesting target for the development of anti-vitiligo agents. A series of novel isoxazole chalcone derivatives have been designed, synthesized, and evaluated for biological activities by our group. Among them, derivative 1-(4-((3-phenylisoxazol-5-yl)methoxy)phenyl)-3-phenylprop-2-en-1-one (PMPP) was identified as a potent tyrosinase activator with better activity and lower toxicity than the positive control 8-methoxypsoralen (8-MOP) in this study. Further investigations revealed that Akt and GSK3β were the signaling pathways involved in the hyperpigmentation of PMPP. Overall, these studies may provide a convenient and novel approach for the further development of anti-vitiligo agents.

## 1. Introduction

Vitiligo is an idiopathic, progressive, cosmetic disfigurement of skin due to depigmentation that starts after birth [1]. It affects approximately 0.5% to 1% of the population, and there is no difference in the rate of occurrence according to skin type or race [2]. In spite of recent findings implicating that vitiligo is linked with genetic, immune, and oxidative stress factors, its pathogenesis mechanism is still enigmatic [3]. So far, most studies have thought that the disease mainly results from the destruction of the melanocyte and the obstruction of melanin synthesis [4,5].

Melanogenesis is a physiological process in response to UV exposure. In mammals, melanogenesis is directly regulated by three enzymes, tyrosinase (TYR), tyrosinase-related protein-1 (TRP-1), and TRP-2 [6]. TYR is the primary enzyme in melanogenesis that catalyzes two different chemical reactions: the hydroxylation of tyrosine to produce 3,4-dihydroxyphenylalanine (DOPA) and the oxidation of DOPA to DOPA quinone [7]. TRP-1 oxidizes 5,6-dihydroxyindole-2-carboxylic acid (DHICA) to indole-5,6-quinone-2-carboxylic acid in mice, but not in humans, whereas TRP-2 catalyzes the rearrangement of DOPA chrome to DHICA [8]. Microphthalmia-associated transcription factor (MITF) is known to be the master regulator of melanocyte differentiation, pigmentation, proliferation, and survival [9]. In addition, it is a major transcription factor regulating TYR, TRP-1, and TRP-2 expression [10]. Decreased MITF expression induces the downregulation of differentiation markers and inhibits melanogenesis [11].

Several important signaling pathways have been reported to be involved in melanin synthesis, such as the Wnt/β-catenin signal pathway. When this signal pathway is not activated, β-catenin is phosphorylated by a multiple-protein complex comprising axin, adenomatous polyposis coli (APC), glycogen synthase kinase 3β (GSK3β), and casein kinase 1 (CK1), and then affected by ubiquitin and degradated through ubiquitin proteasomes [12]. On the contrary, when the Wnt pathway is activated by the interactions of Wnt 1, Wnt3a, and Wnt8 with frizzled receptors and low-density lipoprotein receptor-related protein (Lrp) 5/6 co-receptors [13], GSK3β is negatively regulated. Thereafter, cytoplasmic β-catenin will translocate into the nucleus and bind to the promoter of MITF together with LEF1, resulting in the transcriptional activation of MITF [14,15,16]. Another signaling pathway involved in melanogenesis regulation is phosphatidylinositol-3-kinase (PI3K)/Akt signaling. The activation of PI3K/Akt induces melanin synthesis through the upregulation of MITF, tyrosinase, and the TRPs [17].

**Figure 1:**
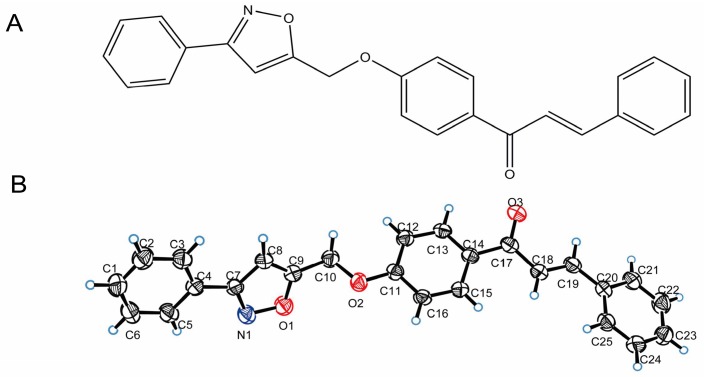
Chemical and crystal structure of PMPP. (**A**) Chemical structure of PMPP; (**B**) Crystal structure of PMPP.

Kaliziri (*Vernohia anthelmintica* (L.) Willd.) is a kind of traditional Uyghur medicinal plant growing only in the high altitude localities of southern Xinjiang and limited regions of Pakistan and India. Its fruit extract has been widely used for treating vitiligo. As one of the most popular Uyghur medicines, Kaliziri was initially recorded in “Yao Yong Zong Ku” around 300 years ago [18]. Some significant flavonoid compounds isolated from this plant have been proved to play a major role in depigmentation treatment [19,20,21]. However, few flavonoids as activators of tyrosinase have been reported. Our research team has been dedicated to studies on the drug in the treatment of vitiligo for years [18,22,23,24]. Recently, a new series of isoxazole chalcone derivatives were designed and synthesized by our research group [23], and evaluated for their functional effects on tyrosinase (data not provided) and melanin synthesis in murine B16 cells. In consideration of the generally low cytotoxicities of these compounds, we further screened them from the two aspects of the structure-activity relationship and biological activity, and identified one chalcone derivative, named 1-(4-((3-phenylisoxazol-5-yl)methoxy)phenyl)-3-phenylprop-2-en-1-one (PMPP) (compound 12 in Ref. [23]) (Figure 1), for further study. Although PMPP was not the stimulator of melanin synthesis with the most potential in these derivatives, it was found to be a promising candidate compound with a stable activating effect on both melanin synthesis and tyrosinase activity. In this study, we evaluated the activity of PMPP on melanogenesis and provided evidence showing that it activates TYR activity and melanin content via upregulating MITF expression depending on the activation of Akt phosphorylation and GSK3β phosphorylation and the induction of β-catenin accumulation in B16 cells.

## 2. Results

### 2.1. Morphological Changes of Melanoma Cells Induced by PMPP

**Figure 2:**
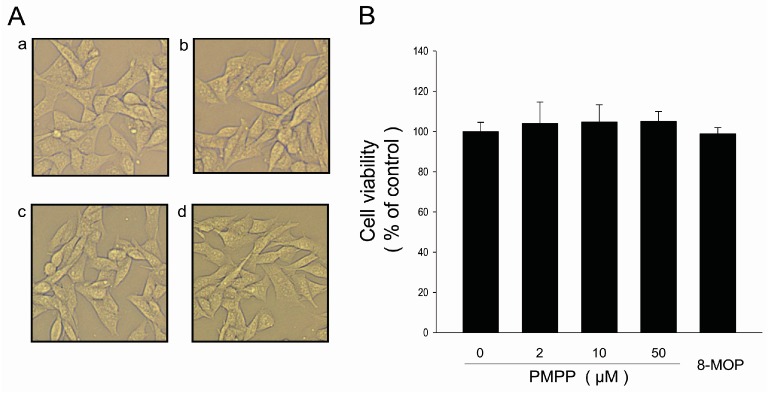
Effects of PMPP on cell morphology and cell viability. (**A**) Effects of PMPP on cell morphology. B16 cells were treated with 0.1% DMSO as a vehicle (a) or with PMPP at 2 (b), 10 (c), and 50 μM (d) for 24 h. Cell morphology was observed under a microscope. Magnification, ×200; (**B**) Effects of various concentrations of PMPP on cell viability. B16 melanoma cells were exposed to various concentrations of PMPP (0, 2, 10, and 50 μM) for 24 h. Cell viability was measured by a CCK-8 assay. The data are shown as the means ± SD; *n* = 3.

Our results showed that murine melanoma B16 cells treated with PMPP for 24 h did not induce any changes in cell morphology and viability when compared with untreated cells (Figure 2) Thus, PMPP concentrations at 0–50 μM are suitable for further evaluating the effects of PMPP on tyrosinase activity and melanin synthesis.

### 2.2. Treatment with PMPP Stimulates Tyrosinase Activity and Melanin Content in B16 Cells at Non-Cytotoxic Concentrations

**Figure 3:**
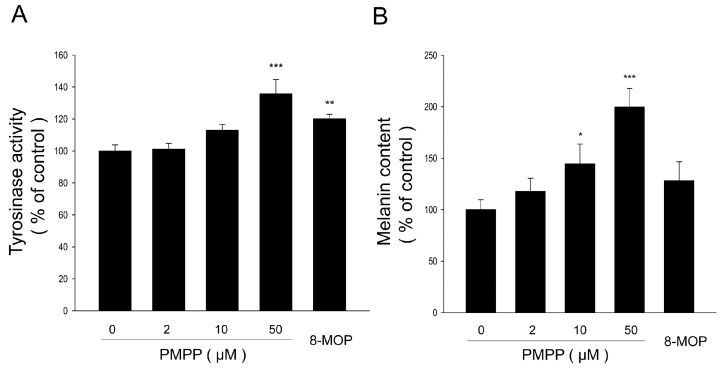
Concentration-dependent effect of PMPP on tyrosinase activity (**A**) and cellular melanin synthesis (**B**) in B16 melanoma cells. Cells were treated with 0.1% DMSO as a vehicle or with PMPP at 2, 10, and 50 μM and 50 μM 8-MOP as a positive control. The cells were then analyzed by tyrosinase activity assay (**A**). The data of melanin content is shown (**B**). Each percentage value for treated cells is reported relative to that of 0.1% DMSO cells. The results have been shown as the means ± SD; *n* = 3, * *p* < 0.05, ** *p* < 0.01, *** *p* < 0.001 compared with 0.1% DMSO cells.

Treatment with PMPP demonstrated the increased tyrosinase activity in a concentration-dependent manner. At the same concentration of 50 μM, the tyrosinase activity of PMPP was increased by 1.2-fold compared with 8-MOP (0 μM, 100 ± 3.8%; 2 μM, 101.1 ± 3.7%; 10 μM, 112.9 ± 3.7%; 50 μM, 135.7 ± 9.0%; 8-MOP, 50 μM, 120.1 ± 2.9%) (Figure 3A). Melanogenesis is known to be controlled through an enzymatic cascade that is regulated by tyrosinase [24]. Thus, we also measured the melanin content in B16 melanoma cells. As shown in Figure 3B, the melanin amount showed the same increasing trend in response to PMPP treatment, and the melanin content of PMPP was increased by 1.6-fold compared with 8-MOP at 50 μM (0 μM, 100 ± 9.6%; 2 μM, 117.8 ± 12.7%; 10 μM, 144.4 ± 19.4%; 50 μM, 199.8 ± 18.1%; 8-MOP, 50 μM, 127.9 ± 18.5%).

### 2.3. Effect of PMPP on the Expressions of TRPs

**Figure 4:**
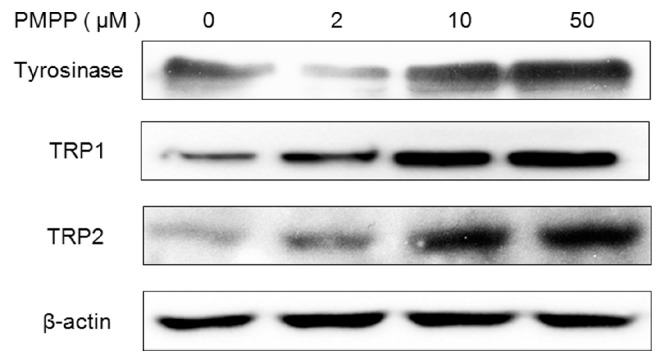
Comparison of Tyrosinase and TRP protein expressions by PMPP. B16 cells were treated with PMPP at 0, 2, 10, and 50 µM for 48 h. Tyrosinase, TRP-1, and TRP-2 protein expressions were detected by Western blotting. The results were normalized against β-actin expression.

Since PMPP increased tyrosinase activity and melanin production, we explored the melanogenic signaling pathway related to the stimulatory activity of PMPP next. After treatment with PMPP, the expressions of melanogenesis-related proteins (MRPs), such as TYR, TRP-1, and TRP-2, were examined by Western blotting. The expression of MRPs was clearly enhanced in a concentration-dependent manner after treatment with PMPP at different concentrations (2, 10, and 50 μM) for 48 h (Figure 4).

### 2.4. PMPP Activates the Wnt Signal Pathway by Regulating the Akt Signal Molecule

**Figure 5:**
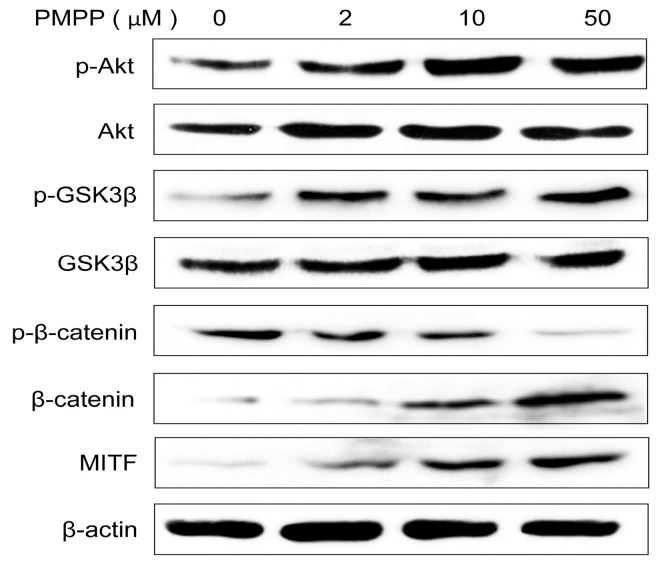
Effects of PMPP on signal transduction proteins that participate in melanogenesis. B16 cells were treated with PMPP at 0, 2, 10, and 50 μM for 48 h, and the phosphorylation and total of Akt, GSK3β, β-catenin, and MITF were measured by Western blotting. Equal protein loading amounts were confirmed by β-actin expression.

Reports have shown that the Wnt signal pathway is closely related to melanin synthesis [25]. In order to clarify the mechanism by which PMPP activates melanin synthesis, we measured the changes of GSK3β in B16 cells treated with PMPP. As shown in Figure 5, the level of phosphorylation of GSK3β increased after 48 h at different concentrations of PMPP treatment compared with untreated cells. The phosphorylation of β-catenin at ser33 decreased with a concomitant increase of total β-catenin induced by PMPP. Takeda et al. have revealed that phosphorylated GSK3β restores its activity and enhances MITF binding to the tyrosinase promoter, consequently inducing melanogenesis [26]. As expected, our data illustrated that the expression of MITF induced by PMPP treatment also had a significant increase.

**Figure 6:**
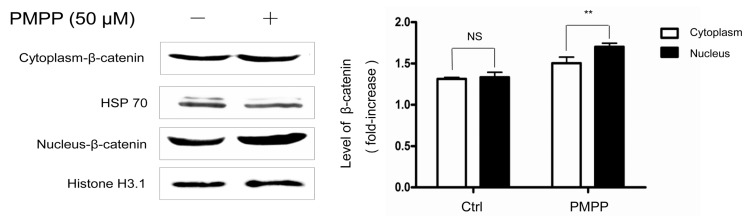
PMPP promotes β-catenin nuclear translocation. B16 cells were treated with PMPP of 50 μM for 12 h, and the expression levels of proteins, including β-catenin in the cytoplasm and nucleus, were detected by using a Western blot. Equal protein loading amounts were confirmed by HSP 70 expression in the cytoplasm and Histone H3.1 expression in the nucleus. Data from the densitometric scanning of band intensities obtained from three separate experiments are presented as means ± SD. NS: No statistical difference, ** *p* < 0.01 compared with untreated cells.

The PI3K/Akt signaling pathway is claimed to be closely relevant to GSK3β in previous research [27,28,29], and it is believed that activated Akt can phosphorylate GSK3β at ser9, inactivating GSK3β and inhibiting the degradation of β-catenin [30]. Consistent with the literature, our data indicated that PMPP upregulates p-Akt and p-GSK3β (the inactive form of GSK3β) simultaneously in B16 cells. Importantly, the β-catenin content in the nucleus (the active β-catenin) was enhanced obviously after 12 h of PMPP treatment compared with untreated cells, while the content in the cytoplasm did not change (Figure 6). These observations revealed that the Akt signal molecule is directly involved in the Wnt signal pathway of the melanogenesis mediated by PMPP.

### 2.5. Effects of PMPP on the Melanogenesis-Related Signaling Pathways by Specific Inhibitors in B16 Melanoma Cells

Since PMPP could activate the PI3K/Akt and GSK3β signaling pathways, to throw further light on this conclusion, we hypothesized that BIO (a selective inhibitor of GSK3β) and Akt inhibitor IV (a selective inhibitor of Akt) could influence the stimulating effect of PMPP on melanogenesis.

**Figure 7:**
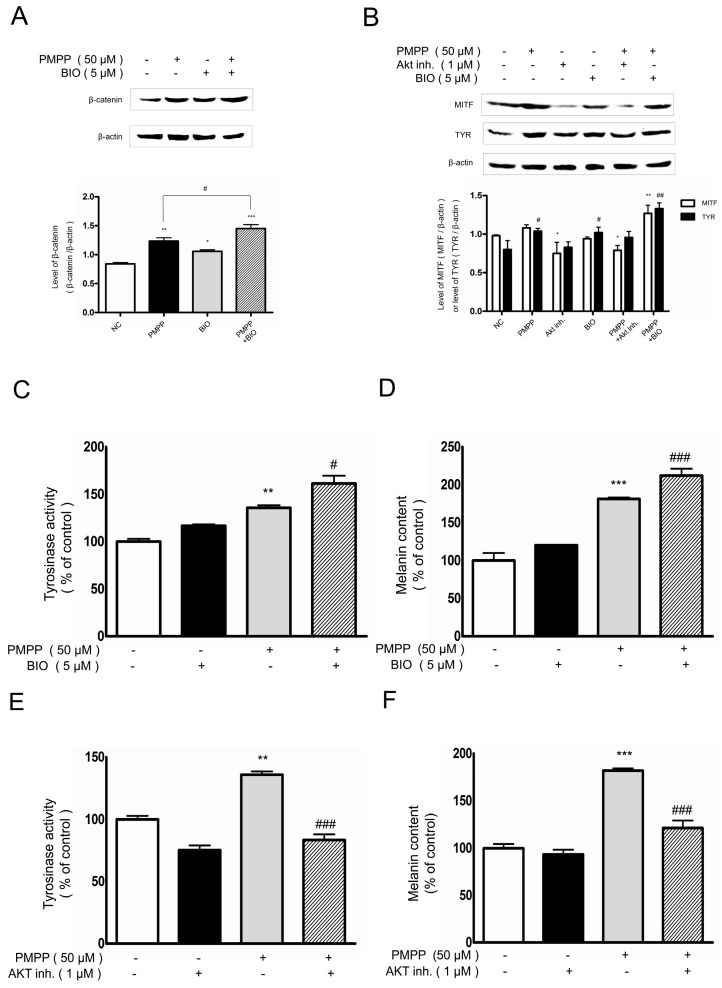
Effects of PMPP on β-catenin, Microphthalmia-associated transcription factor (MITF), tyrosinase (TYR) protein expression and TYR activity, and melanin content in B16 cells treated with GSK3β or Akt inhibitor. (**A**) The GSK3β inhibitor increased PMPP-induced β-catenin expression. β-catenin was detected in B16 cells pre-treated with or without BIO (5 μM) and incubated with PMPP (50 μM) or not. Data from the densitometric scanning of band intensities obtained from three separate experiments are presented as means ± SD. * *p* < 0.05, ** *p* < 0.01, *** *p* < 0.001 versus untreated cells, ^#^ *p* < 0.05 versus PMPP alone; (**B**) Effects of inhibitors on PMPP-induced MITF and TYR expression. The expressions of MITF and TYR were determined by Western blot in B16 cells treated with PMPP or not in the presence or absence of BIO (5 μM) or Akt inhibitor IV (1 μM) for 48 h. Data from the densitometric scanning of band intensities obtained from three separate experiments are presented as means ± SD. * *p* < 0.05, ** *p* < 0.01 versus MITF control, ^#^ *p* < 0.05, ^##^ *p* < 0.01 versus TYR control. GSK3β inhibitor enhanced PMPP-induced TYR activity (**C**) and melanin content (**D**). The Akt inhibitor suppressed PMPP-induced TYR activity (**E**) and melanin content (**F**). B16 cells pre-incubated with inhibitors (BIO 5 μM, AKT inhibitor IV 1 μM) were further incubated with 50 μM PMPP, then followed by an additional incubation for 24 h (tyrosinase activity) or 48 h (melanin content). Each percentage value in the treated cells was calculated with respect to that in the untreated cells. Values are expressed as the mean ± SD of three separate experiments. ** *p* < 0.01, *** *p* < 0.001 compared with control; ^#^ *p* < 0.05, ^###^ *p* < 0.001 compared with PMPP stimulation.

According to the literature, BIO makes β-catenin accumulate in cells, which results in an increase of TYR activity and melanin content [16]. We examined the possibility that the increased expression levels of MITF and tyrosinase proteins induced by PMPP may be restored by BIO. In our study, B16 cells were treated with PMPP in the presence or absence of BIO for 2 days. A Western blot analysis of B16 cells treated with a GSK3β inhibitor confirmed β-catenin stimulation (Figure 7A). As a consequence of this stimulation, an enhancement of MITF and TYR protein expression activated by BIO was observed (Figure 7B). As expected, the GSK3β inhibitor also significantly induced PMPP-triggered tyrosinase activity and melanin synthesis (Figure 7C,D). These results suggest the possibility that the PMPP induced β-catenin accumulation in a GSK3β-dependent manner.

Similarly, we examined the effect of the Akt inhibitor on the expression level of MITF and TYR in B16 cells co-treated with PMPP (Figure 7B). The data are consistent with Figure 7E,F; the Akt inhibitor reversed the upregulation of MITF and TYR in B16 cells co-treated with PMPP. Besides that, tyrosinase activity and melanin content were distinctly aborted. Taken together, the data gathered in this study demonstrated that the induction of melanogenesis by PMPP is regulated by the specific inhibitors of melanogenic signaling pathways, which involves the PI3K/Akt and GSK3β signaling pathways.

## 3. Discussion

Although spontaneous re-pigmentation occurs in 25% of patients, the treatment options for vitiligo are still limited. In the 1930s, 8-methoxypsoralen (8-MOP) and 5-methoxypsoralen (5-MOP) were isolated from *Psoralea corylifolia* L., [31,32]. Later, other psoralens, such as 4,5,8-trimethylpsoralen (TMP), were synthesized as well. Among them, 8-MOP is considered to be a better therapeutic agent against vitiligo when considering low doses and toxicity. Studies [33,34] have reported that 8-MOP leads to dramatic increases in melanin production through activating the protein kinase A and/or protein kinase C signaling pathways. However, 8-MOP clinical therapy is accompanied by some undesirable side effects, such as gene mutation, skin phototoxicity, and a risk of skin cancer [35,36]. In this regard, there is an increasing need to develop safer and more effective substitutions for treating skin de-pigmentation.

Chalcones are a ubiquitous group of natural organic compounds concentrated in licorice, safflower, psoralens, and other medicinal plants. They are naturally occurring intermediates of flavonoid compounds [37] and exhibit numerous biological activities, such as antileishmanial [38,39], antituberculosis [40], analgesic, antipyretic, antibacterial, antifungal [41], and anti-HIV [42] activities. Chalcones are readily synthesized by a variety of different methods, and their chemical features allow for great structural and functional versatility [43,44], which makes chalcone an interesting target for the development of anti-vitiligo agents.

Our team has reported the synthesis and biological evaluation of a series of isoxazole chalcone derivatives. In the process of further screening, the safety and efficacy of these derivatives were equally valued. In consideration of the structure-activity relationship of these derivatives, including the type, number, toxicity, and activity of substituting groups, some derivatives with stable chemical structures and a low toxicity of substituents were selected. Next, these derivatives were set up in a concentration gradient of 0, 2, 10, 50 µM for further investigation. The results indicated that only PMPP with low toxicity showed a stable activating effect in enhancing both melanin synthesis and tyrosinase activity in a concentration-dependent manner. So, we selected this derivative as a promising candidate for further study. The melanin content and tyrosinase activity increased by 1.6-fold and 1.2-fold, respectively, in B16 cells treated with PMPP compared with 8-MOP treated controls in our research. Furthermore, PMPP significantly activated the expression of melanogenic proteins such as TYR, TRP-1, and TRP-2.

Several signaling pathways involved in melanin synthesis include the mitogen-activated protein kinases (MAPKs) signaling pathway, the cAMP signaling pathway, the PI3K/Akt signaling pathway, and the Wnt/β-catenin signal pathway [45,46]. While other signaling pathways had been effectively shut out by our preliminary experiment, the Akt and Wnt signal pathways were demonstrated to be closely related to PMPP-induced melanin synthesis. In order to unravel the molecular mechanism, we measured the changes of GSK3β in B16 cells treated with PMPP. The results showed that PMPP increased intracellular p-GSK3β expression, resulting in the accumulation of the β-catenin in the cytoplasm, which translocated into the nucleus and combined with the promoter of MITF, contributing to the upregulation of MITF. Here, GSK3β inhibition was associated with a marked increase in the levels of β-catenin protein in the B16 cells, confirming that the Wnt pathway was activated (Figure 7A). The effects on the melanogenesis of PMPP and the phosphorylation of GSK3β demonstrated in our research are consistent with the role of the Wnt signaling pathway in hyperpigmentation. The inactivation of GSK3β can be mediated by some kinases, including Akt, PKA, PKC, and p90rsk via phosphorylation at ser9 in GSK3β [47]. In this study, the phosphorylation of Akt was clearly enhanced for 48 h compared with cells treated with 0.1% DMSO only, which suggested that the PMPP-mediated elevation of GSK3β might be Akt-dependent. To further confirm that both the Akt and GSK3β signaling factors are responsible for PMPP-induced activation effects on melanogenesis, inhibitors (Akt inhibitor IV and BIO) were co-incubated with PMPP in B16 cells. It was noteworthy that the PMPP-stimulated melanin content and tyrosinase activity were clearly influenced by the inhibitors of Akt and GSK3β. As expected, GSK3β inhibition promoted β-catenin accumulation and the expression level of MITF and TYR in B16 cells co-treated with PMPP, while the Akt inhibitor reversed these effects.

## 4. Materials and Methods

### 4.1. Sample Preparation

The synthetic procedure of the target compound was based on our team’s previous report. It was characterized by ^1^H-NMR, ^13^C-NMR, IR, and HRMS (ESI) [23]. The single crystals of the compounds (CCDC No. 1577360) used for the X-ray diffraction analysis were obtained by slow evaporation of an acetone-ethanol (*V*/*V* = 1:3) mixed solution at room temperature. Yield 85%, white solid, m.p. 131–133 °C. PMPP was dissolved in DMSO and stored at −20 °C as a stock solution (50 mM). A voucher specimen (No. CAM-D201512) was deposited in The Key Laboratory of Plant Resources and Chemistry of Arid Zone, Xinjiang Technical Institute of Physics and Chemistry, Chinese Academy of Sciences.

### 4.2. Reagents and Antibodies

DMSO (D8418) was bought from Sigma-Aldrich (St. Louis, MO, USA). A cell counting kit (FC101) was purchased from TransGen Biotechnology (Beijing, China). A nuclear and cytoplasmic protein extraction kit (P0027) and HSP70 antibody (AF0189) were bought from Beyotime Biotechnology (Shanghai, China). Akt inhibitor IV (989841-49-7) was bought from EMD Biosciences (San Diego, CA, USA). BIO (EY0580) was purchased from AMQUAR Biology (Shanghai, China). AKT (#5373), p-AKT^s473^ (#9271), GSK3β (#9832), p-GSK3β^s9^ (#9323), β-catenin (#8480), and β-actin (#3700) antibodies were purchased from Cell Signaling Technology (Danvers, MA, USA). p-β-catenin^s33^ (sc-16743-R), antibodies against TYR (sc-7833), TRP-1 (sc-25543), and TRP-2 (sc-25544) were bought from Santa Cruz Technology (Dallas, TX, USA). Anti-MITF (#2650908) antibody was purchased from Millipore (Billerica, MA, USA). Anti-mouse (sc-2970), anti-goat (sc-2020), and anti-rabbit (sc-2365) IgG antibodies (horseradish peroxidase conjugated) were purchased from Santa Cruz Biotechnology (Dallas, TX, USA). Histone H3.1 antibody (#21137) was purchased from SAB Biotechnology (College Park, MD, USA).

### 4.3. Cell Culture

The murine B16 melanoma cell line was acquired from the Chinese Academy of Sciences (Beijing, China). Cells were cultured in Dulbecco’s modified Eagle’s medium (DMEM, Gibco Life Technologies, Waltham, MA, USA) supplemented with 10% heat-inactivated fetal bovine serum (FBS), penicillin G (100 U/mL), and streptomycin (100 mg/mL) (Gibco-BRL, Grand Island, NY, USA) at 37 °C in a humidified atmosphere of 5% CO_2_.

### 4.4. Cell Morphology and Cell Viability Measurement

Cell morphology was examined under a LEICA DMI8 microscope (LEICA microsystems CMS GmbH, Wetzlar, Germany). The general viability of the cultured cells was assayed by adding CCK-8 solution. Broadly speaking, B16 cells were plated in 96-well dishes at a density of 5 × 10^3^ cells per well and allowed to adhere for 24 h. Test samples were added, and the cells were incubated for 24 h. After discarding the culture medium of the cells, 10 μL of CCK-8 solution was added into each well and the cells were incubated at 37 °C for another 2 h. The absorbance was determined at 450 nm using a Spectra Max M5 (Molecular Devices company, Sunnyvale, CA, USA). The absorbance of cells without treatment was regarded as 100% of cell survival. Each treatment was performed in triplicate, and each experiment was repeated three times.

### 4.5. Tyrosinase Activity Assay

Cellular tyrosinase activity was estimated by measuring the rate of L-DOPA oxidation. Briefly, B16 cells were seeded in a six-well plate at a density of 2 × 10^5^ cells per well and allowed to attach for 24 h. Cultured B16 cells were incubated in the absence or presence of PMPP for 24 h, and the cells were washed with ice-cold PBS twice, lysed with 1% Triton X-100 solution containing 1% sodium deoxycholate for 30 min at −80 °C, and each lysate was centrifuged at 12,000× *g* for 15 min to obtain the supernatant. Ninety microliters (90 μL) of the supernatant was added to 10 μL of freshly prepared substrate solution (10 mM L-DOPA) in a well of a 96-well plate. Then, the cells were incubated at 37 °C in the dark for 60 min and the optical densities were measured at 490 nm using an ELISA reader. Activity was measured using the following formula: tyrosinase activity (%) = (OD_490_ of sample/OD_490_ of control) × 100.

### 4.6. Melanin Measurement

B16 cells were seeded in a six-well plate (2 × 10^5^ cells/well) and allowed to attach for 24 h. Cultured B16 cells were incubated in the absence or presence of PMPP for 48 h and then washed with ice-cold PBS twice. After the cells were lysed according to a previously described method with a slight modification [8], each lysate (150 μL) was put in a 96-well microplate and measured spectro-photometrically at 405 nm by a multi-plate reader. The protein concentration of each sample was determined by a BCA Protein Assay Kit (Biomed, Beijing, China). The melanin content was normalized to the cellular protein concentration. The percentage value of the PMPP-treated cells was calculated with respect to the untreated cells. Each treatment was performed in triplicate, and each experiment was repeated three times.

### 4.7. Western Blot Analysis

The PMPP-treated cells were lysed in cold RIPA Lysis buffer (pH 7.4) containing protease and a protease inhibitor cocktail (1 M 4-nitrophenyl phosphate disodium salt hexahydrate (PNPP), 1 M sodium fluoride (NaF), 10 mM phenylmethanesulfonyl fluoride (PMSF), 100 mM benzamidine, 100 mM d,l-Dithiothreitol (DTT), and 200 mM sodium orthovanadate (OV)) for 30 min on ice. The whole-cell lysate was collected and regarded as a protein sample. Its concentration was measured by a BCA Protein Assay Kit. Sixty micrograms (60 µg) of individual protein samples were separated by 12% SDS polyacrylamide gels and transferred onto polyvinylidene fluoride (PVDF) membranes (Merck Millipore Ltd., Billerica, MA, USA). Membrane blocking was performed with a 5% skim milk solution for 1 h, and the samples were then incubated with the primary antibodies at 4 °C overnight. Equal loading was assessed using anti-β-actin antibody to normalize the amounts of total protein. After three washes with TBST buffer containing 50 mM Tris (pH 7.6), 150 mM NaCl, and 0.2% Tween-20, the membranes were incubated with horseradish peroxidase-conjugated secondary antibodies at a dilution of 1:2000 for 1 h at room temperature. The targeted proteins were detected by ECL Western blotting detection reagents (GE Healthcare, Parramatta, NSW, Australia) and visualized using the ChemiDoc MP Imaging system (Bio-Rad Laboratories, Inc., Hercules, CA, USA). The Western bolt assay results reported here are representative of at least three experiments.

### 4.8. Statistical Analysis

Results were expressed as the means ± SD, and the statistical analysis was performed by one-way ANOVA followed by a Tukey post hoc test for multiple comparison tests. A two-tailed value of *p* < 0.05 was considered to be a significant difference.

## 5. Conclusions

**Figure 8:**
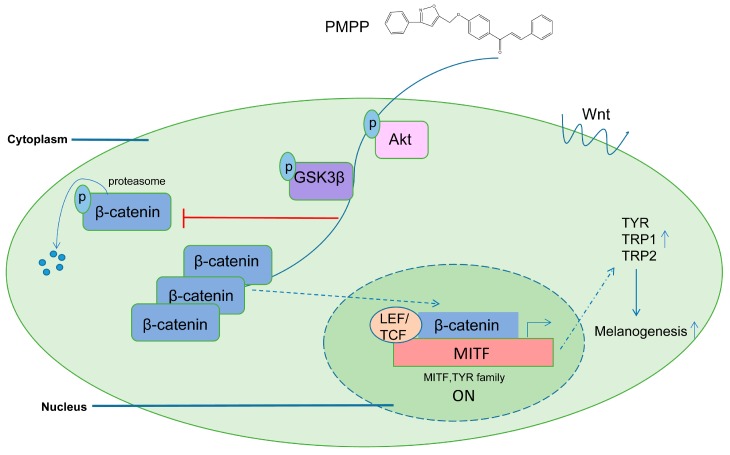
The possible mechanism of PMPP activating melanin content in B16 cells. PMPP increases the phosphorylation of Akt, and thus probably increases the non-activated GSK3β, which inhibits the phosphorylation of β-catenin and ceases its degradation via proteasomes. β-catenin accumulating in cytoplasm up-regulates MITF content through promoting transcription with LEF/TCF (Lymphoidenhancer factor/T cell factor) after coming into nucleus. The augmentation of β-catenin probably leads to the increases in the transcription of MITF and the TYR family and the subsequent induction of melanin synthesis.

To sum up, in this study, PMPP is recognized as a promising candidate compound with an activating effect on melanin synthesis and tyrosinase activity that is much better than the positive control 8-MOP in this study. We evaluated the activity of PMPP on melanogenesis and provided solid evidence showing that it can effectively induce melanogenesis in B16 cells by increasing the phosphorylation of GSK3β through Akt activation, promoting the accumulation of β-catenin, inducing β-catenin into the nucleus, and increasing the expression of MITF and the TYR family (Figure 8). These results unravel a molecular function for the isoxazole chalcone derivative PMPP in melanogenesis, and may provide some new approaches for seeking promising activators of tyrosinase based on chemically synthesized compounds. Currently, further studies of animal experiment on vitiligo model mice are under way and more studies are required to assess the safety and efficacy of PMPP for clinical use.
